# Supplementary material for: Negative feedback via RSK modulates Erk‐dependent progression from naïve pluripotency
Source: EMBO Rep. 2018 Jun 12;19(8):e45642. doi: 10.15252/embr.201745642 (PMC6073214; doi:10.15252/embr.201745642)
Supplement: Supplementary file 1 — Expanded View Figures PDF [file EMBR-19-e45642-s001.pdf]

Expanded View Figures

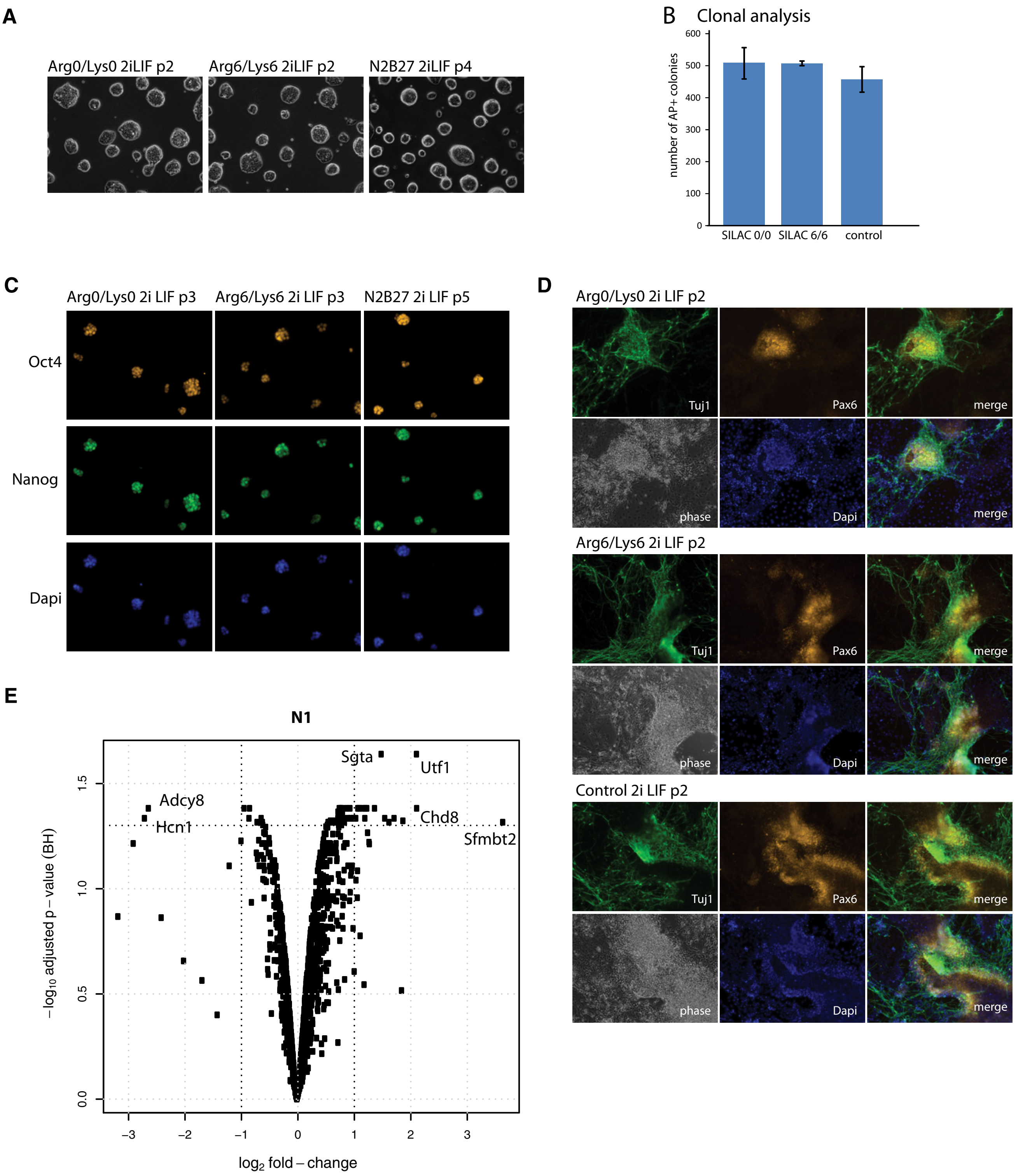

Figure EV1.

 © 2018 The Authors

Figure EV1. Characterisation of SILAC-labelled ES cells. Related to Fig 1.

Cells were grown in 2iLIF for two passages before transfer into light (Arg0/Lys0) and heavy (Arg6/Lys6) SILAC medium.

- A Phase contrast images of self-renewing ES cell colonies in defined SILAC medium supplemented with 2iLIF. Arg0 = normal arginine; Lys0 = normal lysine; Arg6 = heavy arginine containing 6 × ^{13}C, Lys6 = heavy lysine containing 6 × ^{13}C. 20× magnification.
- B Histogram showing number of alkaline phosphatase-positive colonies formed from single cells deposited in 2iLIF after labelling for 2 passages in light (SILAC 0/0), heavy (SILAC 6/6) or full N2B27 (without Neurobasal)/2iLIF (control) medium. Mean and SD shown; n = 2.
- C Immunostaining of SILAC-labelled cells with Oct4 and Nanog antibodies after three passages in SILAC medium. 20× magnification.
- D Immunostaining of SILAC-labelled ES cells with Pax6 and Tuj1 antibodies on day 9 of culture in N2B27. Note: Arg6/Lys6 cells were treated with Chiron and LIF for 24 h before clonal analysis and gene expression profiling. p, passage. 20× magnification.
- E Volcano blot illustrating fold changes and statistical significance for identified phosphorylated peptides in the nuclei fraction (N1). Results are from protein identifications in three independent eperiments.

Figure EV2. RSK knockdown, knockout and rescue. Related to Fig 2.

- A ES cells were transfected with RSK isoform-specific siRNAs in 2i and gene expression analysed 48 h after transfection. 1 = RSK1; 2 = RSK2; 3 = RSK3; 4 = RSK4. The Ct values are calculated relative to *Gapdh* and normalised to scrambled siRNA. Mean and SD shown; n = 2.
- B RSK gene structure. Introns are shown in green and exons in grey. Red arrows indicate exon targeted by gRNAs.
- C Genomic PCR strategy to identify potential candidate clones. For each gene, a three-primer PCR was carried out. Wild-type clones resulted in two bands (larger one—red–red primer pairing, and smaller one—red–blue primer pairing). An indel would result in reduced binding of the internal primer (blue) and amplification of only the large fragment.
- D Rps6ka2 (RSK3) expression analysis in mutant lines. Expression is relative to *Gapdh* and normalised to RGd2 parental line. Mean and SD shown; n = 2.
- E Rps6ka1 (RSK1) expression analysis in mutant and rescue lines. Expression is relative to *Gapdh* and normalised to RGd2 parental line. Mean and SD shown; n = 2.
- F Immunoblot analysis of RSK1 and pERK1/2 in mutant cells after stable transfection with an RSK1 expression vector. Lysates were collected 1 h after 2i/LIF withdrawal. Control is a clone picked in parallel to RSK1*23 which was not targeted by gRNAs.
- G RSK1*23 and parental cells were exchanged from 2iLIF into N2B27 for 22 h and cell lysates at indicated time points. Expression of pERK and ERK was detected by immunoblotting. Second biological replicate shown. Expression of pERK and ERK was quantified using Fiji and the pERK/ERK ratio plotted (right pannel). Grey areas highlight pERK/ERK peaks in the first replicate.
- H Expression of pERK target genes in control and RSK1*23 cell lines after withdrawal of 2i/LIF. Expression relative to *Actb*. Mean and SD shown; n = 2.

Source data are available online for this figure.

© 2018 The Authors

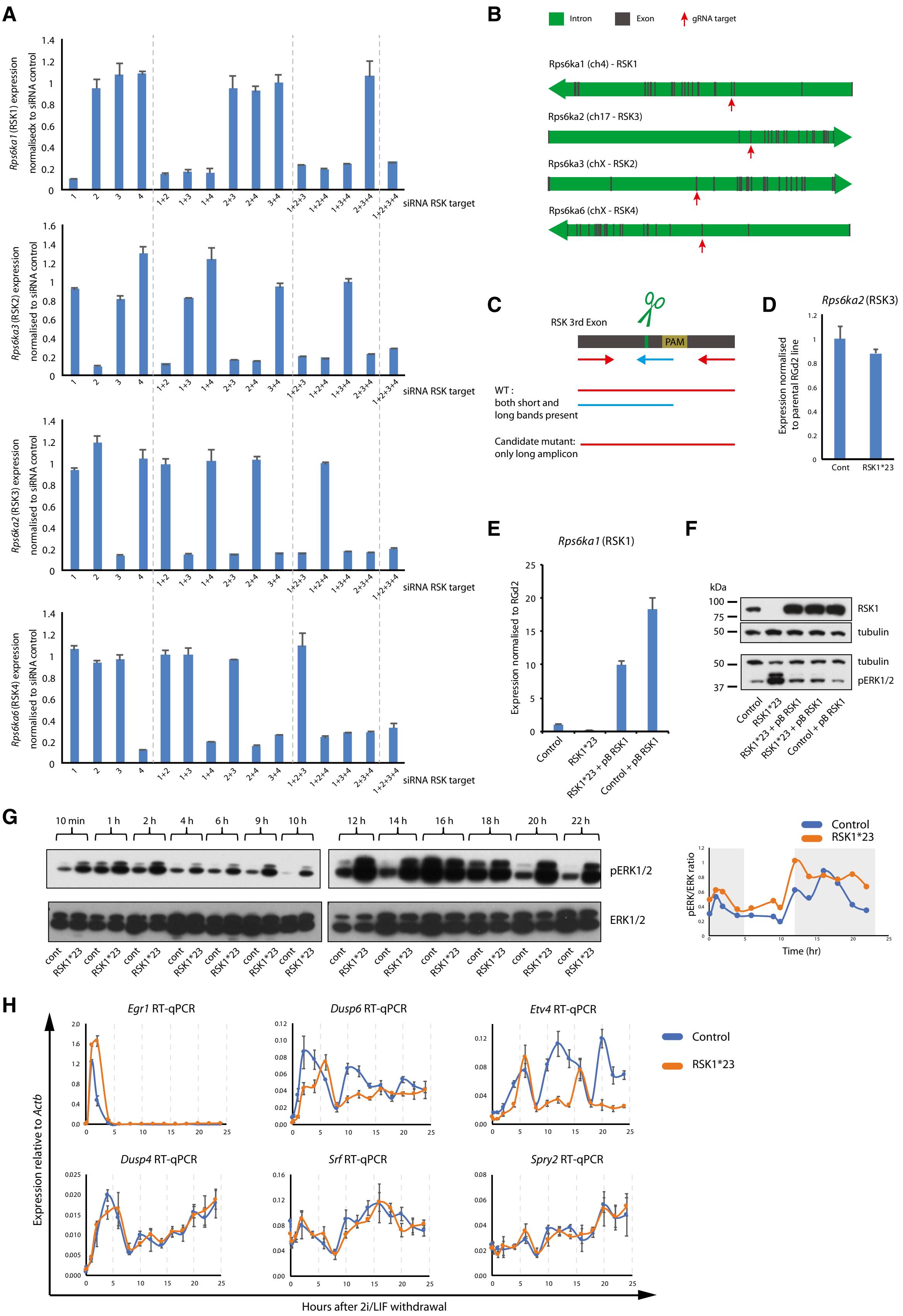

Figure EV2.

© 2018 The Authors

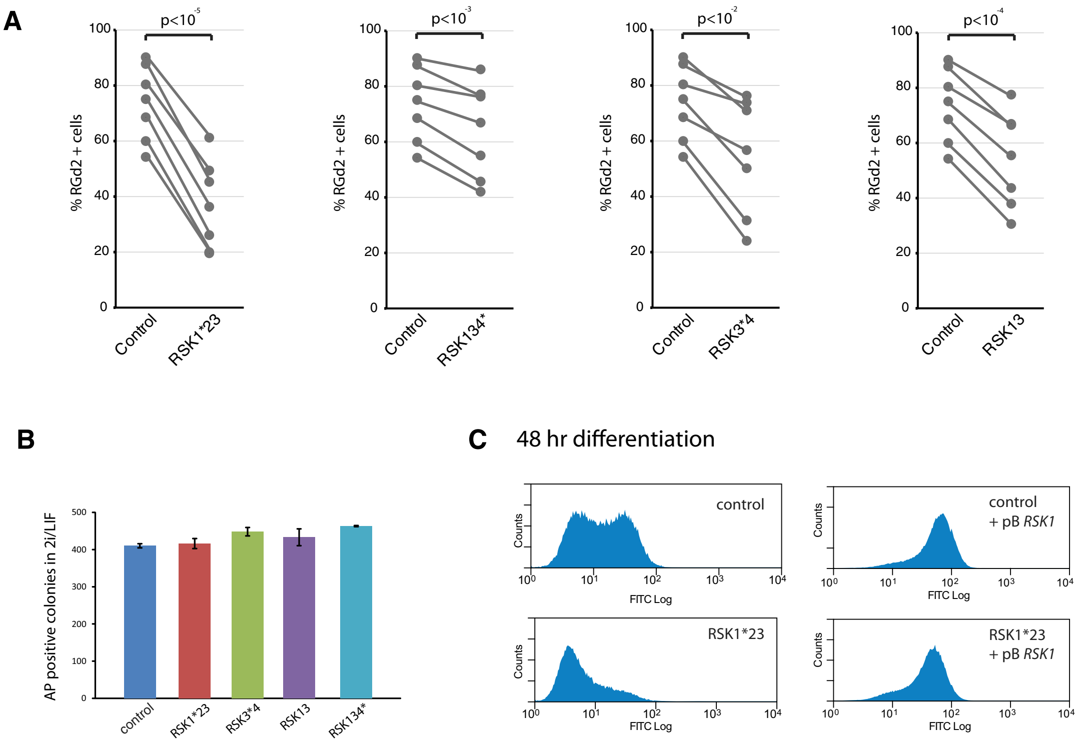

Figure EV3. Colony formation efficiency of *RSK* mutant cells and GFP expression after RSK1 rescue. Related to Fig 3.

A Quantification of percentage of RGd2-positive cells across experiments, at different time points. Lines indicate experiments plated and analysed at the same time. $n = 7$ across different time points; paired t-test shown.

B One thousand cells were plated per 6 wells in 2iLIF and colonies stained for AP on day 5. Columns show numbers of AP^+ colonies. Mean and SD shown; $n = 2$.

C Flow cytometry analysis of *RSK1*-transfected RSK1*23 cells. Cells were withdrawn from 2iLIF for 48 h and analysed for GFP. Control is a clone picked in parallel to RSK1*23 which was not targeted by gRNAs.

© 2018 The Authors

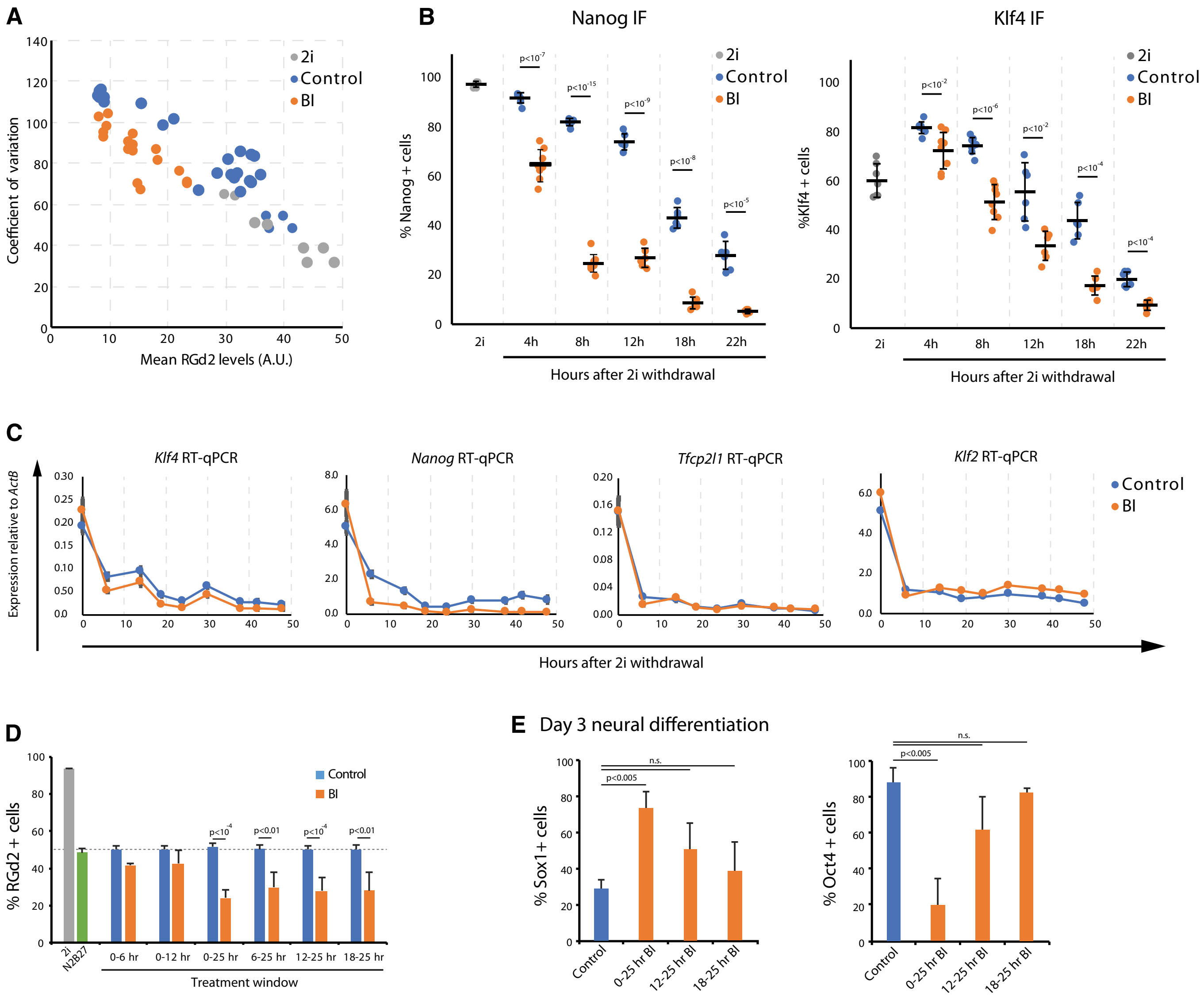

Figure EV4. Treatment with BI at different time points. Related to Fig 5.

A Mean RGd2 fluorescence levels plotted against the coefficient of variation for cells in 2i, or differentiated in BI or control for different time points.

B Percentage of Nanog-positive and Klf4-positive cells over the differentiation time-course in the presence of BI or control. Mean and SD shown; $n = 3$; unpaired two-tailed Student's t-test.

C Bulk mRNA expression of naïve pluripotency factors in a differentiation experiment in the presence or absence of BI. Expression is relative to *Actb.* Mean and SD shown; $n = 2$.

D Percentage of RGd2-positive cells when BI is added at different intervals, relative to control, DMSO-treated, cells. Mean and SD shown; $n = 3$; unpaired two-tailed Student's t-test.

E Percentage of Sox1- and Oct4-positive cells on day 3 of neural differentiation when BI was added at different intervals. Mean and SD shown; $n = 3$; unpaired two-tailed Student's t-test.

© 2018 The Authors
