# Supplementary figures and images for: Negative feedback via RSK modulates Erk‐dependent progression from naïve pluripotency
Source: EMBO Rep. 2018 Jun 12;19(8):e45642. doi: 10.15252/embr.201745642 (PMC6073214; doi:10.15252/embr.201745642)

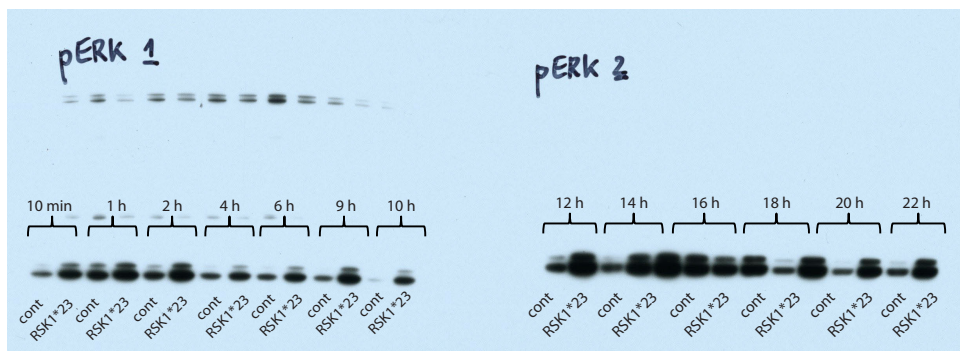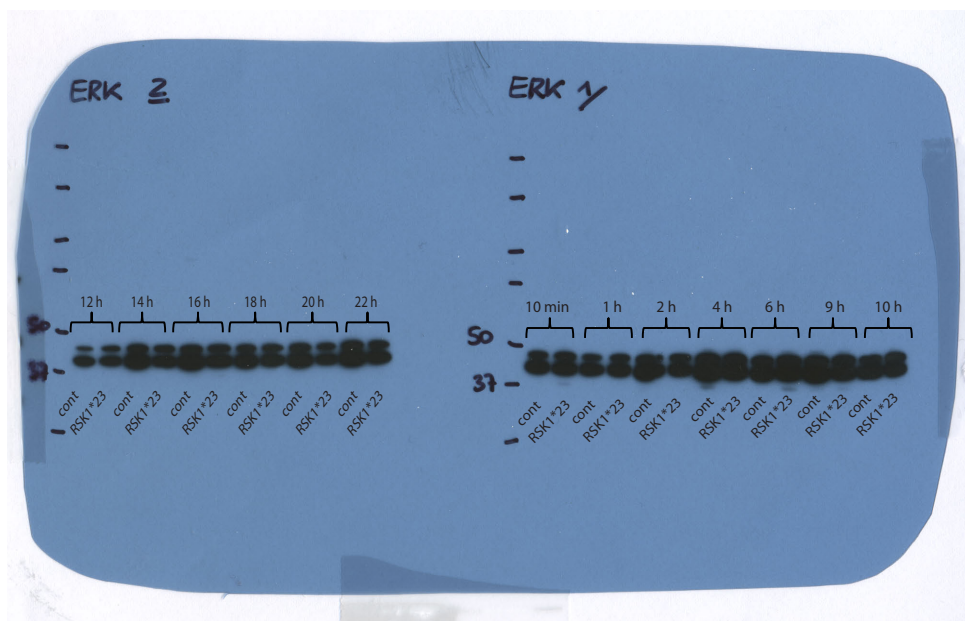

Related to Figure EV2G

Supplement: Supplementary file 7 — Source Data for Expanded View [file EMBR-19-e45642-s011.zip › embr201745642-sup-0011-SDataFigEV2.pdf]

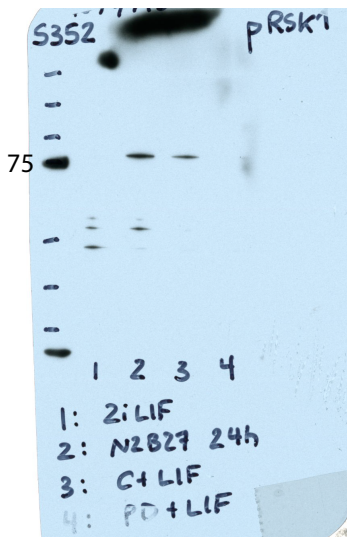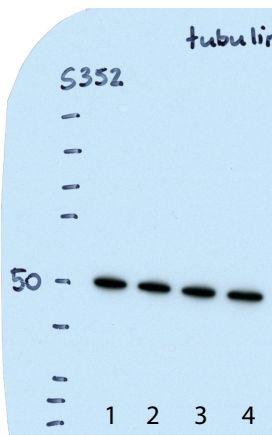

Related to Figure 1D

Samples:

- 1: 2iLIF
- 2: 24hr N2B27
- 3: 24hr CHLIF
- 4: 24h PDLIF

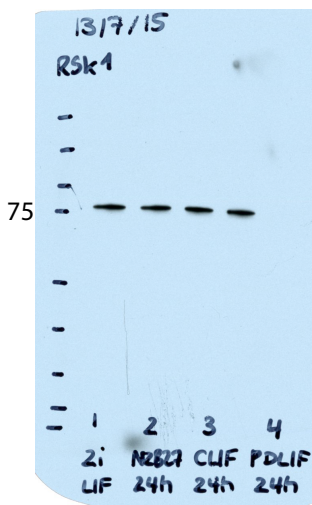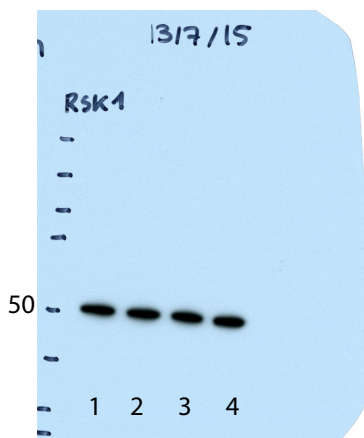

Supplement: Supplementary file 9 — Source Data for Figure 1 [file EMBR-19-e45642-s007.pdf]

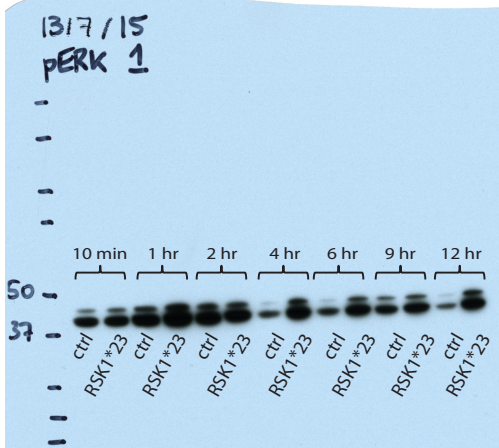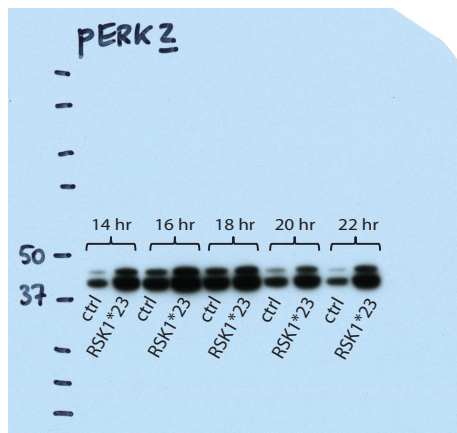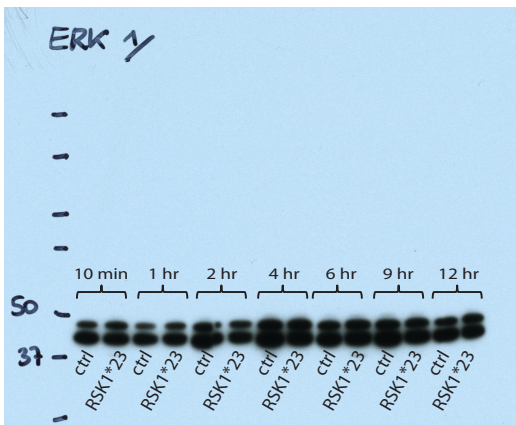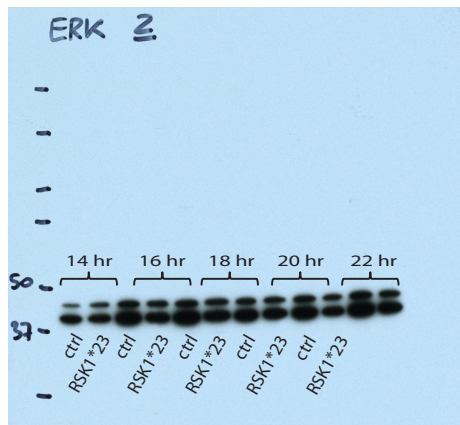

Related to Figure 2D

Supplement: Supplementary file 10 — Source Data for Figure 2 [file EMBR-19-e45642-s008.zip › embr201745642-sup-0006-SDataFig2D.pdf]

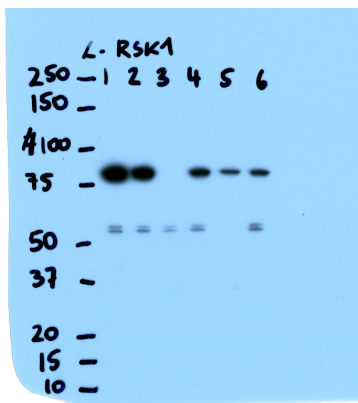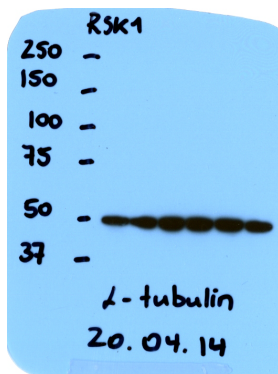

Related to Figure 2C

- 1: RGd2 (parental)
- 2: Control
- 3: RSK1\*23
- 4: RSK134\*
- 5: RSK3\*4
- 6: RSK13

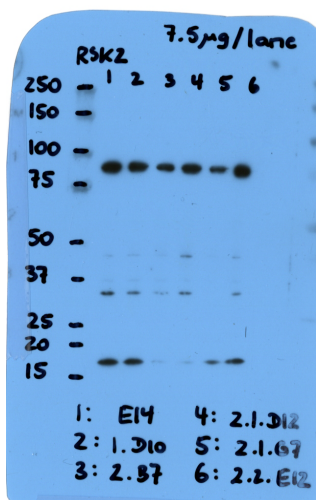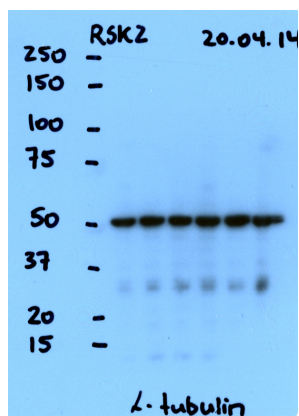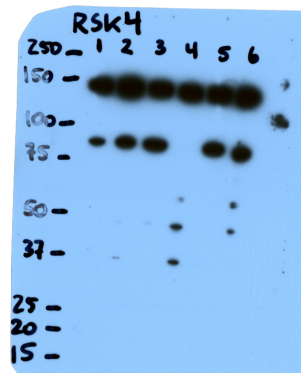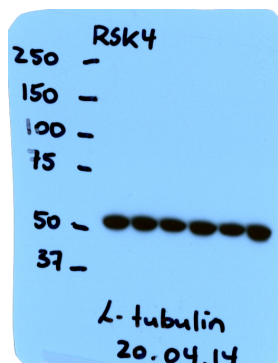

Supplement: Supplementary file 10 — Source Data for Figure 2 [file EMBR-19-e45642-s008.zip › embr201745642-sup-0005-SDataFig2C.pdf]

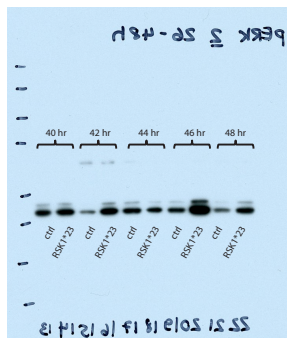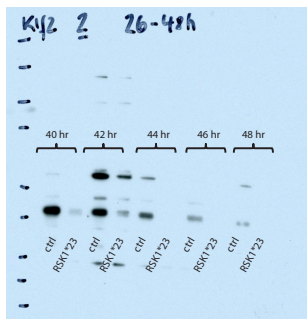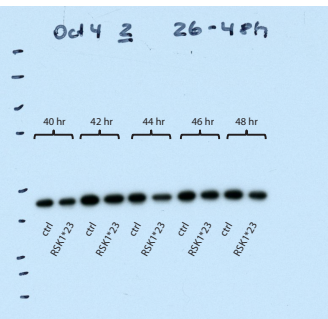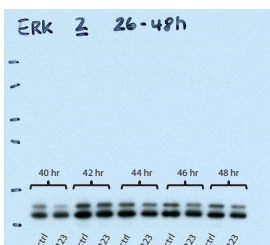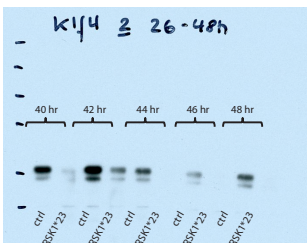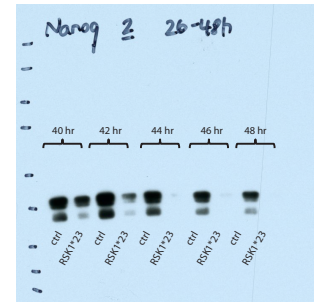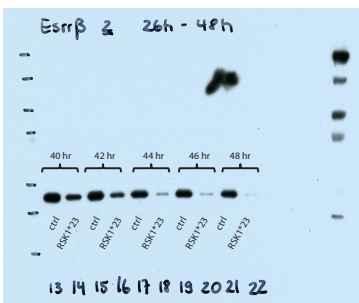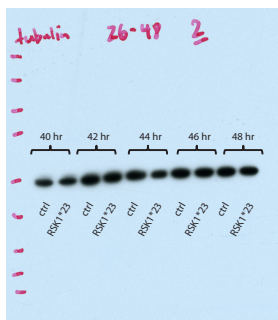

Related to Figure 4A

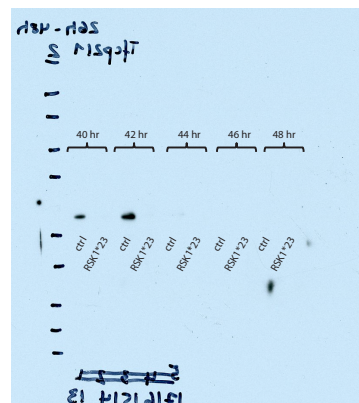

Supplement: Supplementary file 11 — Source Data for Figure 4 [file EMBR-19-e45642-s009.pdf]

Related to Figure 5A

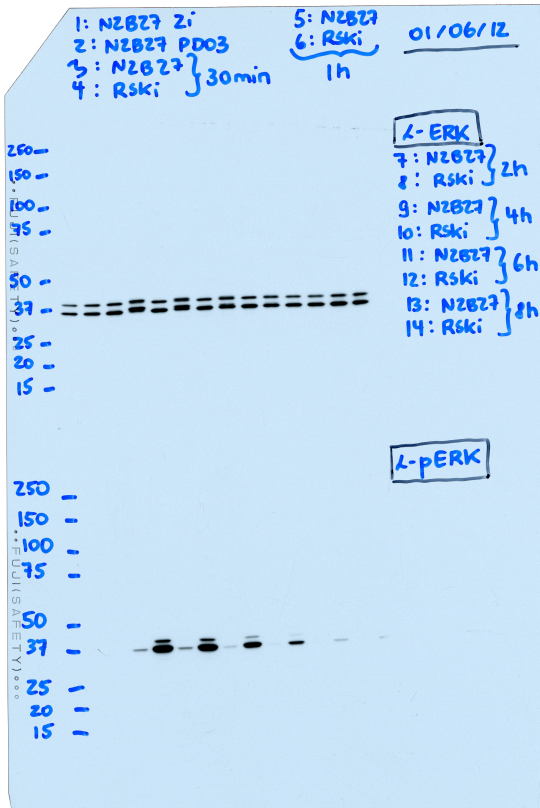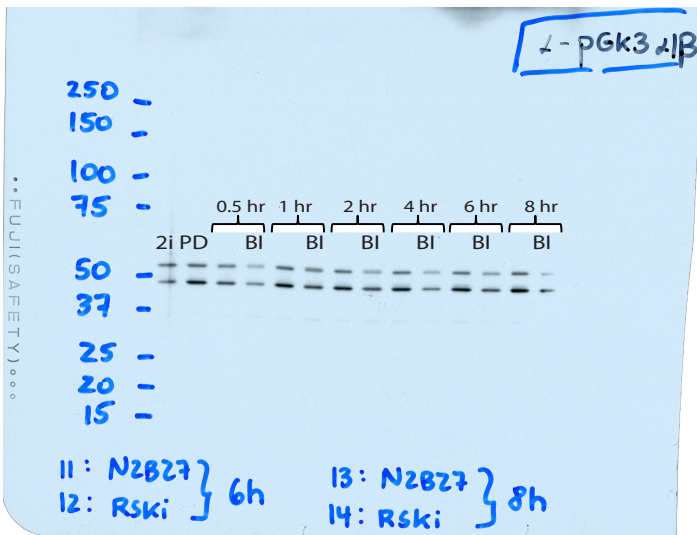

Supplement: Supplementary file 12 — Source Data for Figure 5 [file EMBR-19-e45642-s010.pdf]
