# Supplementary material for: Negative feedback via RSK modulates Erk‐dependent progression from naïve pluripotency
Source: EMBO Rep. 2018 Jun 12;19(8):e45642. doi: 10.15252/embr.201745642 (PMC6073214; doi:10.15252/embr.201745642)
Supplement: Supplementary file 10 — Source Data for Figure 2 [file EMBR-19-e45642-s008.zip › embr201745642-sup-0003-SDataFig2A.pdf]

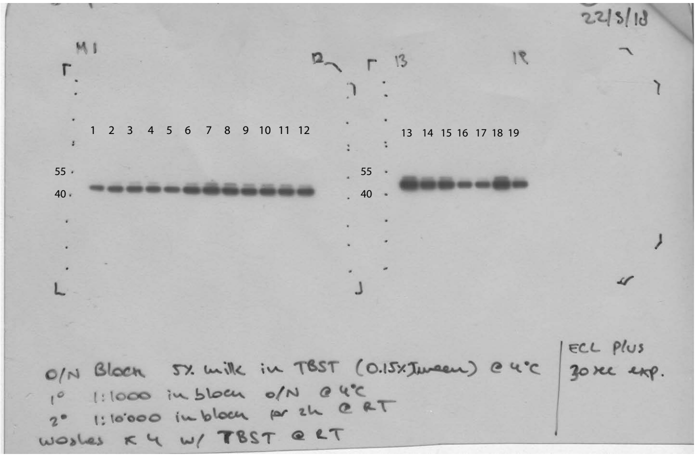

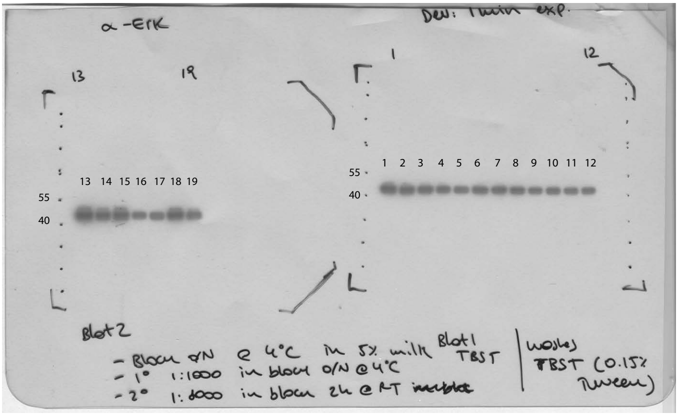

Related to Figure 2A

Samples:
1: RSK1 siRNA
2: RSK2 siRNA
3: RSK3 siRNA
4: RSK4 siRNA
5: Control siRNA
6: RSK1+2 siRNA
7: RSK1+3 siRNA
8: RSK1+4 siRNA
8: RSK2+3 siRNA
10: RSK2+4 siRNA
11: RSK3+4 siRNA
12: Control siRNA
13: RSK1+2+3 siRNA
14: RSK1+2+4 siRNA
15: RSK1+3+4 siRNA
16: RSK2+3+4 siRNA
17: Control siRNA
18: RSK1+2+3+4 siRNA
19: Control siRNA
