# Supplementary material for: Negative feedback via RSK modulates Erk‐dependent progression from naïve pluripotency
Source: EMBO Rep. 2018 Jun 12;19(8):e45642. doi: 10.15252/embr.201745642 (PMC6073214; doi:10.15252/embr.201745642)
Supplement: Supplementary file 10 — Source Data for Figure 2 [file EMBR-19-e45642-s008.zip › embr201745642-sup-0004-SDataFig2B.pdf]

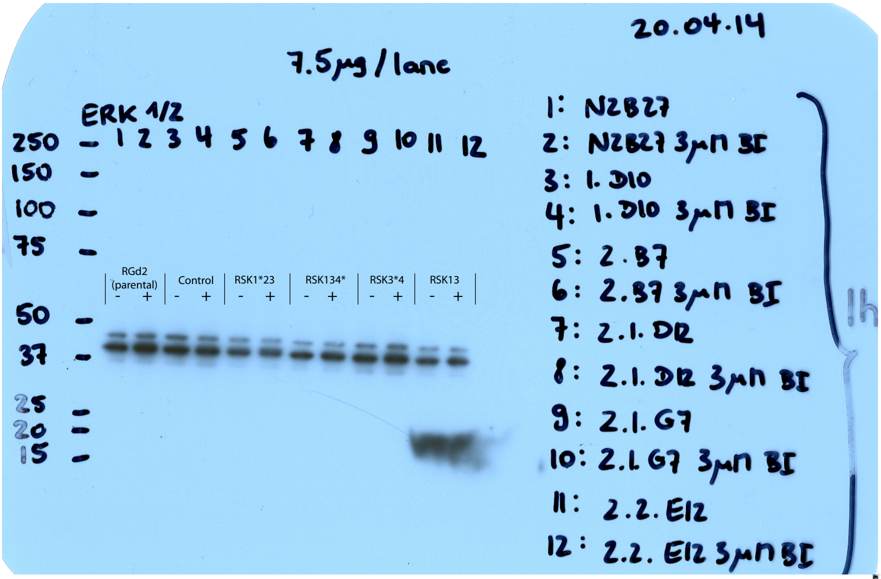

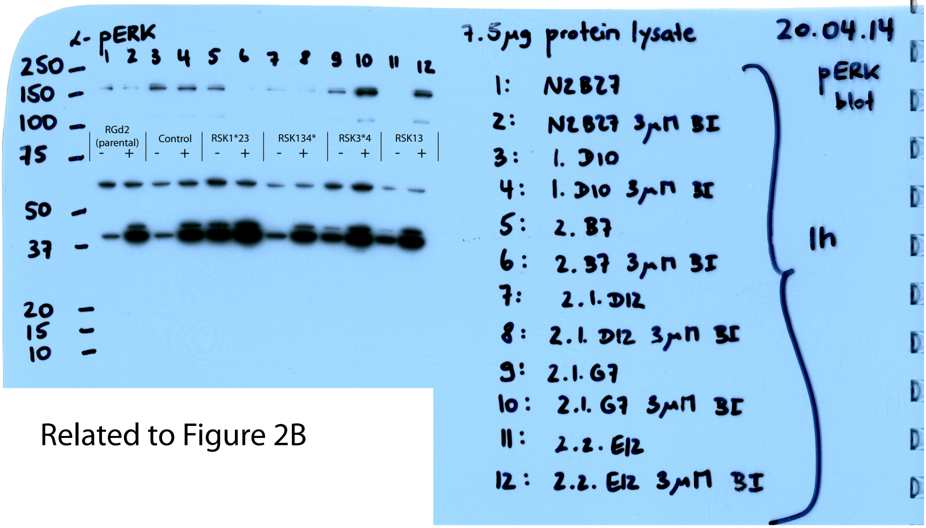

Related to Figure 2B
